# An ecological study on reinfection rates using a large dataset of RT-qPCR tests for SARS-CoV-2 in Santiago of Chile

**DOI:** 10.3389/fpubh.2023.1191377

**Published:** 2023-07-10

**Authors:** Claudio Acuña-Castillo, Carlos Barrera-Avalos, Vivienne C. Bachelet, Luis A. Milla, Ailén Inostroza-Molina, Mabel Vidal, Roberto Luraschi, Eva Vallejos-Vidal, Andrea Mella-Torres, Daniel Valdés, Felipe E. Reyes-López, Mónica Imarai, Patricio Rojas, Ana María Sandino

**Affiliations:** ^1^Departamento de Biología, Facultad de Química y Biología, Universidad de Santiago de Chile, Santiago, Chile; ^2^Centro de Biotecnología Acuícola, Facultad de Química y Biología, Universidad de Santiago de Chile, Santiago, Chile; ^3^Escuela de Medicina, Facultad de Ciencias Médicas, Universidad de Santiago de Chile, Santiago, Chile; ^4^Centro de Investigaciones Biomédicas y Aplicadas, Escuela de Medicina, Facultad de Ciencias Médicas, Universidad de Santiago de Chile, Santiago, Chile; ^5^Centro de Nanociencia y Nanotecnología CEDENNA, Universidad de Santiago de Chile, Santiago, Chile

**Keywords:** COVID-19 pandemic, SARS-CoV-2, vaccines, variants of concern, reinfection

## Abstract

**Introduction:**

As the SARS-CoV-2 continues to evolve, new variants pose a significant threat by potentially overriding the immunity conferred by vaccination and natural infection. This scenario can lead to an upswing in reinfections, amplified baseline epidemic activity, and localized outbreaks. In various global regions, estimates of breakthrough cases associated with the currently circulating viral variants, such as Omicron, have been reported. Nonetheless, specific data on the reinfection rate in Chile still needs to be included.

**Methods:**

Our study has focused on estimating COVID-19 reinfections per wave based on a sample of 578,670 RT-qPCR tests conducted at the University of Santiago of Chile (USACH) from April 2020 to July 2022, encompassing 345,997 individuals.

**Results:**

The analysis reveals that the highest rate of reinfections transpired during the fourth and fifth COVID-19 waves, primarily driven by the Omicron variant. These findings hold despite 80% of the Chilean population receiving complete vaccination under the primary scheme and 60% receiving at least one booster dose. On average, the interval between initial infection and reinfection was found to be 372 days. Interestingly, reinfection incidence was higher in women aged between 30 and 55. Additionally, the viral load during the second infection episode was lower, likely attributed to Chile's high vaccination rate.

**Discussion:**

This study demonstrates that the Omicron variant is behind Chile's highest number of reinfection cases, underscoring its potential for immune evasion. This vital epidemiological information contributes to developing and implementing effective public health policies.

## 1. Introduction

The SARS-CoV-2 is responsible for the current global COVID-19 pandemic, which has resulted in more than 690 million infections and almost 6.8 million deaths worldwide (1). Even though governments and health authorities have implemented numerous measures to prevent or mitigate contagion, the most effective way to control the pandemic is by reducing the number of susceptible individuals in the population. While natural infection by SARS-CoV-2 leads to robust humoral and cellular responses (2), most vaccines can also induce high titers of neutralizing antibodies (3). Various studies on the duration of humoral immunity after a natural infection have reported anti-spike IgG antibodies lasting up to 90 days (4), 6 months (5), and even 11 months (6) after the clinical recovery of the patient.

On the other hand, although immunity from vaccines is still being actively studied, it has been found to last at least 6 months (7). Additionally, new variants of SARS-CoV-2—compared to the ancestral virus—have emerged that can overcome patient immunity due to immune-evading mutations (4, 8). In effect, the Omicron variant, which was declared a variant of concern on November 26, 2022 by the World Health Organization (WHO) (9), has led to a surge of cases in different parts of the world (10, 11), albeit associated with reduced case fatality rates compared to other previous waves of infections, such as the Delta variant waves (12, 13). Omicron subvariants (including BA.1, BA.2, and BA.4/5) have higher immune evasion ability because of additional individual mutations in the S protein (14). Therefore, newer subvariants of Omicron, BQ.1 and BQ.1.1, characterized by increased resistance to neutralizing antibodies, are becoming predominant (15).

The decrease in immunity over time after infection or vaccination and the appearance of new, more elusive variants of SARS-CoV-2 are closely related to reinfections and can result in COVID-19 outbreaks. However, probably, many patients have not been correctly diagnosed, underestimating the official global COVID-19 statistics [revised in (16)]. While numerous isolated reinfections with new variants of SARS-CoV-2 have been reported (17–19), the frequency of reinfection continues to be the subject of the study.

The risk of reinfection has been reported in some studies. The first reports considered reinfections in individuals who previously had COVID-19, which were infrequent (20, 21). The extension of vaccination in different populations and the emergence of new variants during the pandemic became critical factors in determining the incidence of reinfection cases. For example, a study in Malaysia in 2022 reported that the reinfection rate was 6.6 times higher during Omicron circulation than other variants, regardless of the age group, while booster doses decreased the frequency of reinfection compared to sub-optimally vaccinated individuals (22). A study in Iceland in 2022 found that the reinfection rate was 15% among people aged 18–29 during the Omicron wave (23). Multiple reinfections have been reported in South Africa (24), although the incidence of reinfection is higher due to the low percentage of vaccination. However, there are no reports on the rate of reinfection in Chile during the Omicron surge. This country has one of the highest vaccination rates in Latin America [revised in (1)]. This study involves the RT-qPCR tests carried out during the pandemic in the Diagnostic Laboratory of the University of Santiago de Chile (USACH), which reached 578,670 samples of nasopharyngeal swabs (NPSs) from different communes of Santiago de Chile up to July 2022, to analyze the incidence of reinfections. Our results reaffirm the high capacity to evade the immune response presented by the Omicron variant compared to other surges of infections generated by different variants due to the higher prevalence of reinfections under the domain of this variant, even in a population with a complete vaccination schedule ~80%. This report suggests special attention to the increase in reinfection events since they could be related to possible risk groups or the appearance of new, more evasive SARS-CoV-2 viral variants in the population.

## 2. Methods

### 2.1. Study design, sample collection, and COVID-19 diagnosis

The Virology Laboratory of the Universidad de Santiago de Chile (USACH) performed 578,670 RT-qPCR diagnostic tests on nasopharyngeal swab samples (NPSs) from the Central Metropolitan Health Service (CMHS). The CMHS has a catchment population of approximately one and a half million and overseas two hospitals and 17 primary care centers in the western area of the Santiago Metropolitan Region (M.R.). Of these 578,670 RT-QPCR tests, 345,908 corresponded to patients (including positive and negative diagnoses), with 44,181 positive tests from 43,638 patients infected. Diagnostic testing was done from 1 April 2020 to 31 July 2022. The RT-qPCR tests of the M.R. began in the first few days of March 2020. In addition, total RNA from 250 μl of NPSs was extracted as previously described using the Total RNA purification Kit (Norgen Biotek Corp) (25). The detection of SARS-CoV-2 was carried out using the ORF1ab gene probe from TaqMan™ 2019nCoV Assay Kit v1 (Thermo Fisher Scientific, Reference code. A47532) as previously reported by our group (26). We included samples with matching identification numbers to accurately identify RT-qPCR tests from the same patient across different periods. These tests were then grouped based on the Chilean ID number and the date of sample collection. Any discrepancies in dates were individually resolved by cross-referencing the sample ID number with the sample collection date. In case of sex discrepancies, these were manually rectified. Considering the duration of this study extended beyond 2 years, the age reported corresponds to the patient's age at the time of the first test. This methodology provided unambiguous identification of all tests conducted per patient and facilitated the tracking of respective reinfections.

### 2.2. Analysis of infections and reinfections

Five surges were registered in Chile during the study period, which was defined using the data from the M.R. The onset of each wave was determined using the moving average of daily cases, with a window of 7 days to decrease day-to-day variability. The start of each surge was defined when the number of new cases exceeded three standard deviations compared to the previous 3 days. This change in standard deviation coincided with the rate change (first derivative) of the number of cases. The difference in standard deviation could not always be used to determine the end of each surge because it did not always coincide with the change in the slope of the number of points. Moreover, in some cases, the number of points at the end of the surge reached a different level than at the beginning. For those reasons, the end of the wave was defined as the date when the rate of change in the number of cases was closer to zero for at least five consecutive days. Thus, five waves were defined and are shown in Table 1. Reinfections were identified by analyzing wave pairs, with the first infection occurring during the first wave of the couple and reinfection in the second wave. In addition, the second positive test must be at least >90 days after the first positive diagnosis, as previously reported in reinfection studies (27) and according to the Pan American Health Organization (PAHO) criteria (28) and Centers for Disease Control and Prevention (CDC) in 2023 (29). The percentage of reinfection was calculated as the number of reinfections over the number of positive cases in the first surge of the pair. The incidence of reinfection was computed as the number of reinfected patients divided by the cumulative number of persons-day at risk. This follow-up time was calculated as the sum of days from the first positive test to the second positive test or the end of the last surge of the pair. Confidence intervals were computed at 95%.

**Table 1:** Waves of infections in Chile.

	**First wave**	**Second wave**	**Third wave**	**Fourth wave**	**Fifth wave**
Start	22 April 2020	1 December 2020	16 September 2021	25 December 2021	2 May 2022
Final	1 August 2020	1 August 2021	5 December 2021	10 April 2022	31 July 2022
Duration	101 days, 3.4 months	243 days, 8.1 months	80 days, 2.7 months	106 days, 3.5 months	90 days, 3.0 months

To study reinfections independently of the surges (overall reinfection in the study period), a dataset was built with the only criterion being the interval between positive tests >90 days. The percentage of reinfection was calculated as described above. For the incidence rate, the follow-up time for patients with only one positive test was calculated up to the end of the study.

### 2.3. Public data sources

National PCR data from M.R. were obtained from Ministerio de Ciencias Tecnologia Conocimiento e Innovacion (30). SARS-CoV-2 variants were obtained from the GISAID platform (https://gisaid.org/) and Genomic Surveillance Program from Instituto de Salud Publica de Chile (31). Data were analyzed with custom software written in Python.

### 2.4. Ethics

This study was authorized by the Ethics Committee of the University of Santiago of Chile (No. 226/2021) and the Scientific Ethical Committee of the Central Metropolitan Health Service, Ministry of Health, Government of Chile (No. 370/2021), following Chilean legislation.

## 3. Results

### 3.1. Dynamics of epidemiological surges

From 1 April 2020 to 31 July 2022, the COVID-19 diagnostic laboratory at the Universidad de Santiago de Chile (USACH) conducted a total of 578,670 RT-qPCR tests. These tests, performed on nasopharyngeal swab samples, were referred by the Central Metropolitan Health Service. The number of tests correspond to 345,908 distinct individuals. Of these individuals, 43,658 were diagnosed positive for COVID-19, accounting for 44,181 of the positive RT-qPCR tests conducted.

**Figure 1:**
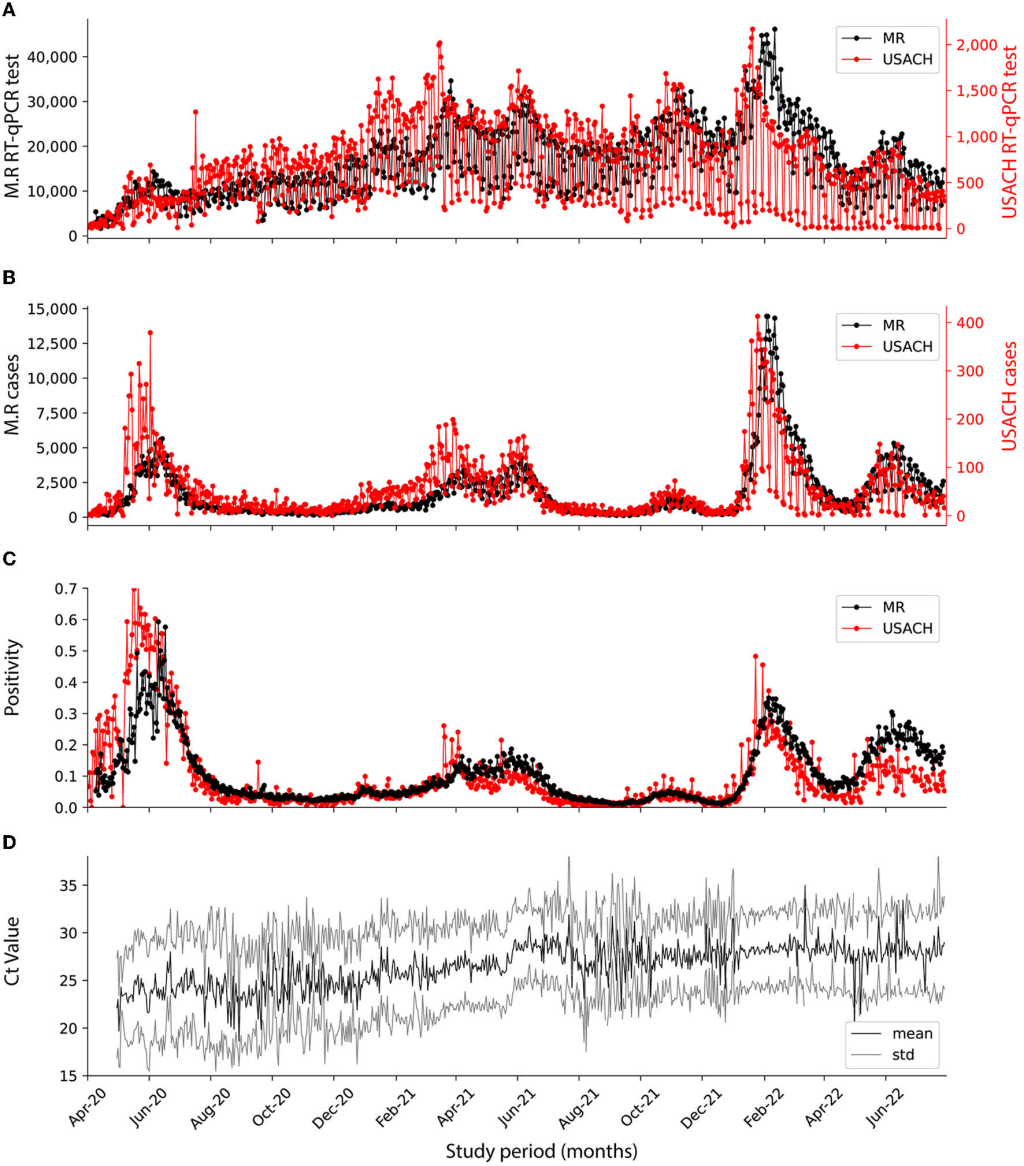
Dynamics of SARS-CoV-2 pandemics in USACH and M.R. at the analyzed period. Comparison of USACH (red) and M.R. (black). **(A)** Number of daily RT-qPCR tests. **(B)** Number of daily cases. **(C)** Daily positivity. **(D)** Daily mean Ct for positive cases (black) with standard deviation (gray).

During the period under review, the quantity of RT-qPCR tests conducted by USACH, represented in red, exhibited significant variability when compared to the entire Metropolitan Region of Santiago, Chile (M.R.), represented in black, as shown in Figure 1A. While the data from the M.R. also demonstrated some degree of weekly variability, it was markedly less pronounced.

By February 2022—the fourth wave—USACH testing capacity peaked at 2,000 tests per day, and by the end of the fifth wave had dropped to 500 tests per day. In the M.R., there was a significant shift in the volume of RT-qPCR tests performed daily between the fourth and fifth waves of the pandemic. During the fourth wave, the M.R. reached a peak of 40,000 tests per day, but this number nearly halved to ~20,000 tests per day during the fifth wave. This represented a considerable reduction of nearly 50% in regional testing at the national level (Figure 1A). The volume of positive cases began notably high at the onset of the pandemic, during the first wave. However, this number subsequently experienced a substantial surge during the fourth wave. This pattern of case incidence was observed similarly in the USACH and the M.R. (Figure 1B). The increased number of infected patients is directly related to increased positivity (defined as positive cases over the total tests analyzed) during the study period. In the first wave, the positivity reached 0.7 for both the overall M.R. testing and the USACH tests. This positivity varied during the pandemic, reaching its lowest value in the third surge, with a relative value of 0.05 for M.R. and USACH. For the fourth surge, the positivity came to 0.4, while, by the end of the fifth surge, it reached 0.25 in USACH data, while for the rest of the wave, the M.R. was close to 0.3 (Figure 1C). The behavior of the results obtained by the USACH was similar to those reported by the total M.R. during the pandemic. This data can closely represent the reinfection incidence in the greater Santiago area.

In April 2020, corresponding to the first wave, the viral Ct values associated with the RT-qPCR diagnosis and viral load were between 22 and 25 (Figure 1D). As the pandemic unfolded, these Ct values increased and reached a maximum close to 27 for the fifth wave of infections (*R*^2^ = 0.833, Supplementary Figure 1), indicating a decrease in the SARS-CoV-2 viral load during the pandemic (32). During the study and within the total number of tests analyzed (578,670), 247,542 patients underwent one RT-qPCR test, 56,007 patients underwent two tests, 42,217 patients underwent more than three tests, and 28 patients underwent up to 45 RT-qPCR tests during the study period (Supplementary Figure 2A). In addition, patients with multiple tests were outnumbered by patients with only one test; hence, the number of tests per patient had a mean of 1.67, a median of 1.0, and a mode of 1.0 test, respectively. Of the total patients examined, 47.7% were women and 45.3% were men, with respective positivity rates of 40.6 and 37.8% (refer to Supplementary Figure 2B). An anomalously high count was observed in patients whose gender could not be ascertained. Throughout the analysis period, the average and median ages of the male patient cohort were 39.4 and 37 years, respectively (Supplementary Figure 2C). In contrast, the female patient cohort had an average age of 41.2 years and a median of 39 years (Supplementary Figure 2D). Among the men who tested positive for COVID-19, the mean age was 39.2 years with a median of 36 years (Supplementary Figure 2E), while for women who tested positive, the average and median ages were 40.3 and 38 years, respectively (Supplementary Figure 2F).

### 3.2. Reinfections

**Figure 2:**
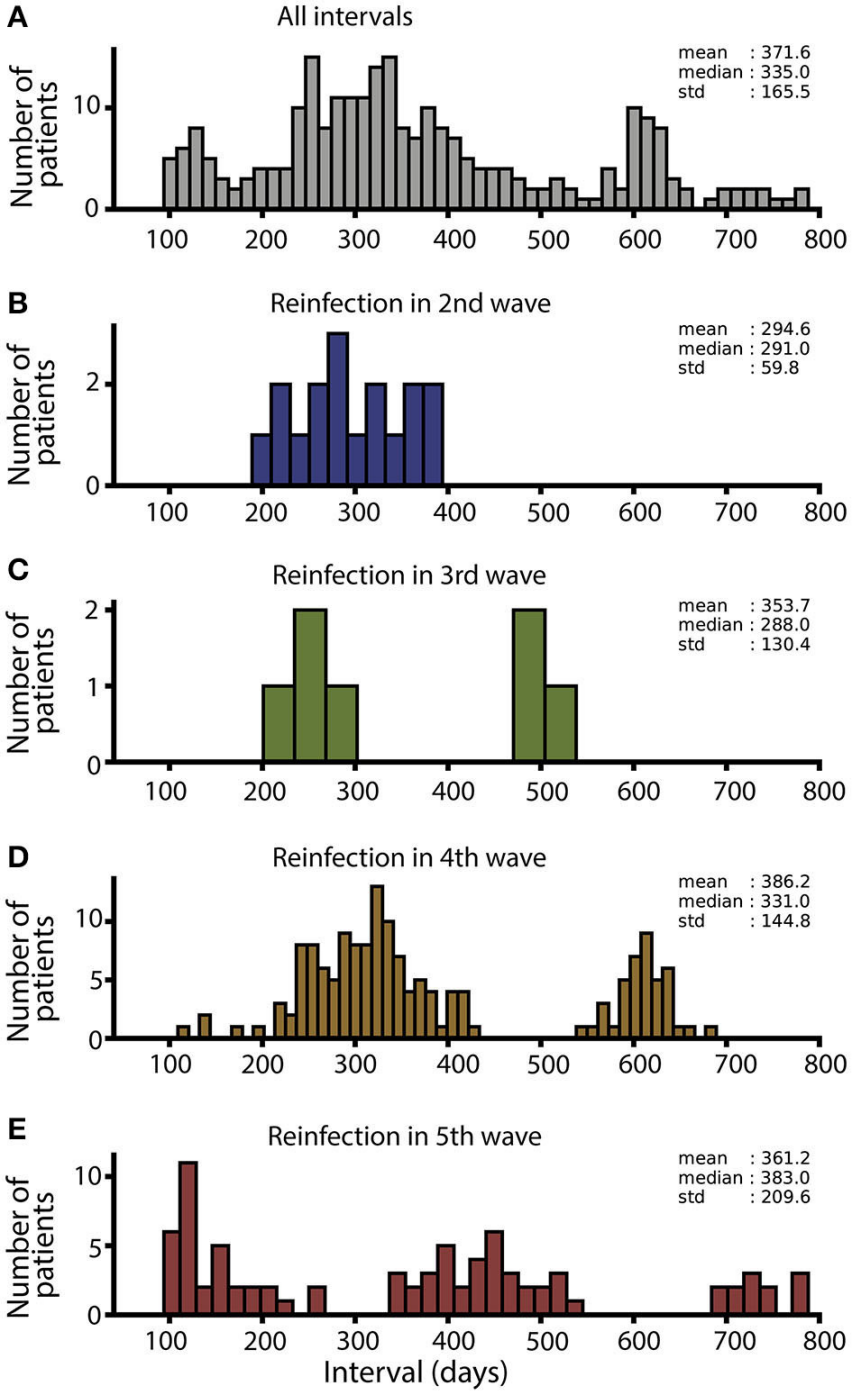
Duration of interval (days) between infections. **(A)** Interval histogram for all reinfections. **(B)** Interval histogram for the 17 reinfections that occurred during the second surge. **(C)** Interval histogram for the seven reinfections that occurred in the third surge. **(D)** Interval histogram for the 159 reinfections that occurred during the fourth surge. **(E)** Interval histogram for the 82 reinfections that occurred in the fifth surge.

To detect potential SARS-CoV-2 reinfections, we focused our analysis on positive cases within paired surge periods. Cases that surfaced during the interim period between surges were not included in this analysis. Reinfections were identified if a patient tested positive during the initial wave of the pair, followed by a second positive test during the subsequent surge. Furthermore, these two test results needed to be spaced apart by more than 90 days, a criterion established based on precedents set in several similar studies (33, 34). With these criteria, 261 reinfections were detected. The period between the two positive tests ranged from 94 to 788 days, with an average duration of 371.6 days and a median of 335 days (Figure 2A). The histogram demonstrating the course of these intervals presents a multimodal distribution due to encompassing intervals from all surge periods. Upon dissecting the data, individual histograms depicting reinfections during the second, third, fourth, and fifth surges reveal diverse populations with varying reinfection intervals (Figures 2B–E, Supplementary Table 1). The shortest reinfection interval occurred in patients who were infected in the first and then in the second wave, with an average time of 294 days before reinfection (Figure 2B). The most extended period between initial infection and reinfection was observed during the fifth wave, with certain patients experiencing over 700 days before a subsequent infection occurred (predominantly with the Omicron variant in the fifth wave). Figure 2 displays histograms of reinfection intervals for each pair of surges, while Supplementary Table 2 provides additional descriptive statistics regarding these durations. Interestingly, the duration between infections tended to be shorter in men (mean = 341.9 days, median = 317.5 days) compared to women (mean = 381.7 days, median = 347 days). The reinfection time intervals for patients in all wave pairs are shown in Supplementary Figure 3.

**Figure 3:**
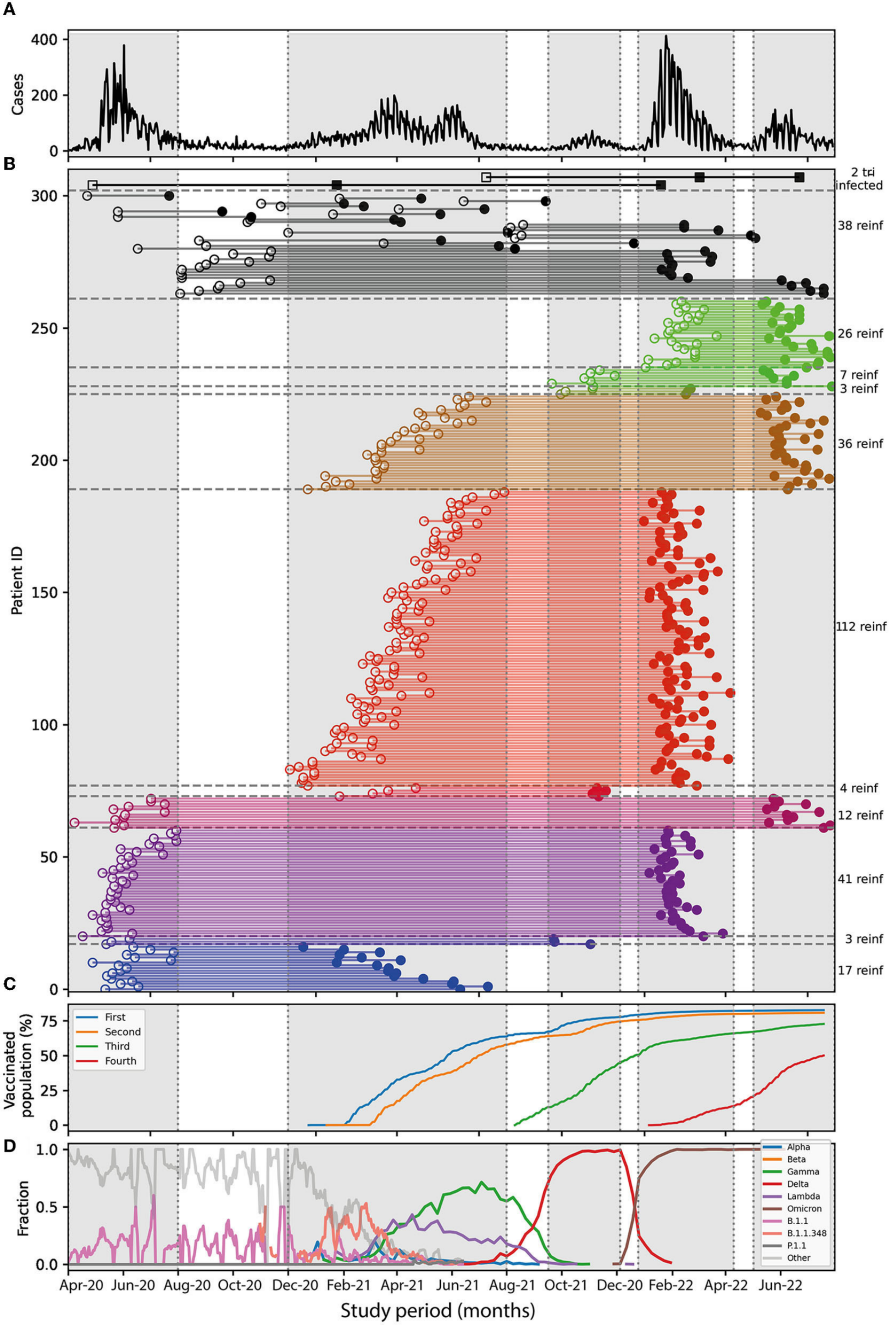
Reinfections between surges. **(A)** Daily cases over time, each surge is enclosed in gray rectangles. **(B)** Interval duration for each reinfected patient, showing first (open circle) and second positive test (filled circle) connected by a colored line representing surges 1–2 (blue), 1–3 (dark blue), 1–4 (purple), 1–5 (magenta), 2–3 (orange), 2–4 (red), 2–5 (golden), 3–4 (pistachio), 3–5 (green), and 4–5 (light green). Reinfections not falling into the surges are represented by black circles. The two cases of tri-infections are shown in squares. To the right, is the number of reinfections for each group. **(C)** Percentage of Chilean population vaccinated with fist dose (blue), second dose (orange), booster dose (green), and second booster (red). **(D)** Time course of the different SARS-CoV-2 variants detected by genome sequencing in Chile. The percentages of the predominance of the variants are the following: First wave: Lambda 48%, others 52%. Second wave: Gamma 62%, Lambda 25, and Alpha 2%. Third wave: Delta 98%, Lambda 0.5%, Gamma 0.25%, and Alpha 0.1%. Fourth wave: Omicron 97% and Delta 3%. The fifth wave, Omicron 100%.

Figure 3 shows the five waves highlighted in gray stripes (Figure 3A) and the reinfection intervals for the 261 patients identified, sorted by date of infection (Figure 3B). For patients infected in the first wave, the highest reinfection rates occurred when the reinfection occurred in the second and fourth waves [0.41 positive tests per 100,000 people (95% CI: 0.213–0.615) and 0.63 (95% CI: 0.43–0.821), respectively, Table 2]. During the second wave, Gamma and Lambda were the most prevalent variants, and during the fourth wave, Omicron was the most prevalent variant (Figure 3D). By the end of the second wave, more than 60% of the Chilean population presented a complete vaccination scheme with two doses (Figure 3C). For patients infected in the second wave, the highest reinfection rate occurred during the fourth wave [2.08 positive tests per 100,000 people (95% CI: 1.687–2.473), Table 2], where the most abundant variant was Omicron (98%, Figure 3D). At the end of the fourth wave, more than 60% of the population had taken two doses and a booster dose (Figure 3C). For patients infected in the third and fourth waves, the highest reinfection rates occurred during the fifth wave [waves 3–4: 0.97 test per 100,000 people (95% CI: 0–2.1), waves 3–5: 1.34 (95% CI 0.327–2.354), and waves 4–5: 1.36 (95% CI: 0.823–1.886), Table 2]. During this wave, the Omicron variant was 100% predominant, and close to 50% of the population had a fourth vaccination, corresponding to the second booster (Figures 3C, D). Overall, the highest incidence rates of reinfection occurred between waves 2–4, 3–4, 3–5, and 4–5, where Omicron was the most abundant variant.

**Table 2:** Reinfections and incidence rate for all pairs if surges.

**Pairs of surges**	**Number of reinfections**	**Number cases**	**% Reinfection**	**CI % reinfection**	**Days follow-up**	**Inc. Day/ 100,000 hab**	**CI Inc. day**
1–2	17	9,723	0.18	(0.092, 0.258)	4,102,245	0.41	(0.213, 0.615)
1–3	3	9,723	0.03	(0.0, 0.066)	5,329,306	0.06	(0.0, 0.121)
1–4	41	9,723	0.42	(0.293, 0.55)	6,551,920	0.63	(0.43, 0.821)
1–5	12	9,723	0.12	(0.054, 0.193)	7,642,998	0.16	(0.066, 0.248)
2–3	4	14,643	0.03	(0.001, 0.054)	3,545,956	0.11	(0.0, 0.226)
2–4	112	14,643	0.77	(0.624, 0.906)	5,383,844	2.08	(1.687, 2.473)
2–5	36	14,643	0.25	(0.166, 0.326)	7,029,365	0.51	(0.341, 0.683)
3–4	3	1,914	0.16	(0.0, 0.334)	307,875	0.97	(0.0, 2.1)
3–5	7	1,914	0.37	(0.095, 0.636)	522,114	1.34	(0.327, 2.354)
4–5	26	11,119	0.23	(0.144, 0.324)	1,919,446	1.36	(0.823, 1.886)

On the other hand, with 100% predominance of the Omicron variant for the fifth surge, close to 50% of the population had a fourth vaccination, corresponding to the second booster. In addition, two patients had a triple infection (Figure 3B). For one, the last infection occurred during the fourth surge and another on the fifth surge, with a predominance of the Omicron variant. However, triple infection only represented 0.8% of all reinfections analyzed in this study.

**Figure 4:**
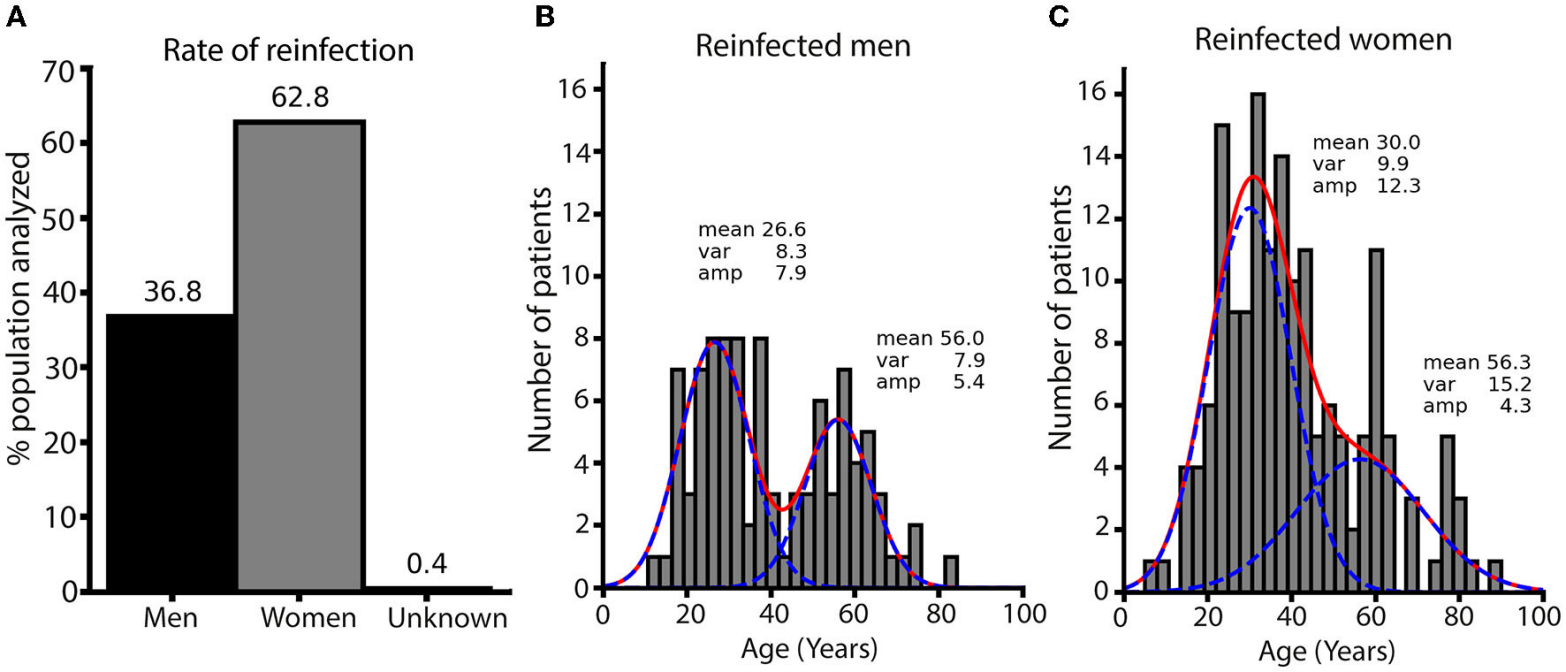
Distribution of age and sex in reinfected patients. **(A)** Percentages of reinfected men and women patients. The numbers above each bar are percentages. **(B)** Histogram of the age distribution for reinfected men. The red line is the fit to two Gaussians, and the blue lines are each Gaussian. **(C)** Histogram of the age distribution for reinfected women. Lines are the same as for men. Both histograms were built with the same range and number of bins to allow the comparison of the count. Fitted parameters are on the top of each Gaussian distribution. The ratio of low/high age calculated from fitted amplitudes is 1.56 for men and 2.82 for women.

Regarding sex differences in the incidence of reinfection, we observed notable differences. Women presented a higher rate of reinfection than men, with 62.8 and 36.8%, respectively (Figure 4A). There is a bimodal age distribution for both sexes; the mean age of reinfected men was 26.6 and 56 years (Figure 4B). While for women, the means were of 30.0 and 56.3 years, respectively. However, women have more reinfections at lower ages than men (Figure 4C). The average period of reinfection in men was 347.35 days, while in women, 385.49, indicating that women in Chile last longer before becoming infected again (Supplementary Figure 4). To quantify these differences, the proportion of patients with lower and higher ages for each group and the amplitudes from each component of the Gaussians were used to obtain the ratio of low/high patients ages. For male patients with positive reinfections, the ratio of low/high ages was ~1.5. However, women reinfected have a ratio of 3.0, meaning a greater number of reinfected young women patients. The most significant differences in the number of men and women occurred in reinfections in the fourth and fifth surges (Supplementary Figure 5A). Higher numbers of women of low age were reinfected between surges 2–4, 2–5, and 4–5, while for surges 1–4 and 1–5, the increase was at all ages (Supplementary Figure 5B). Men were also reinfected at young ages but with fewer events in these last two surges. These data, therefore, indicate that the highest prevalence of reinfection occurred during the predominance of the Omicron variant in women, even in a scenario where the Chilean population had high vaccination rates.

### 3.3. PCR cycle threshold (Ct) value in a reinfection event

**Figure 5:**
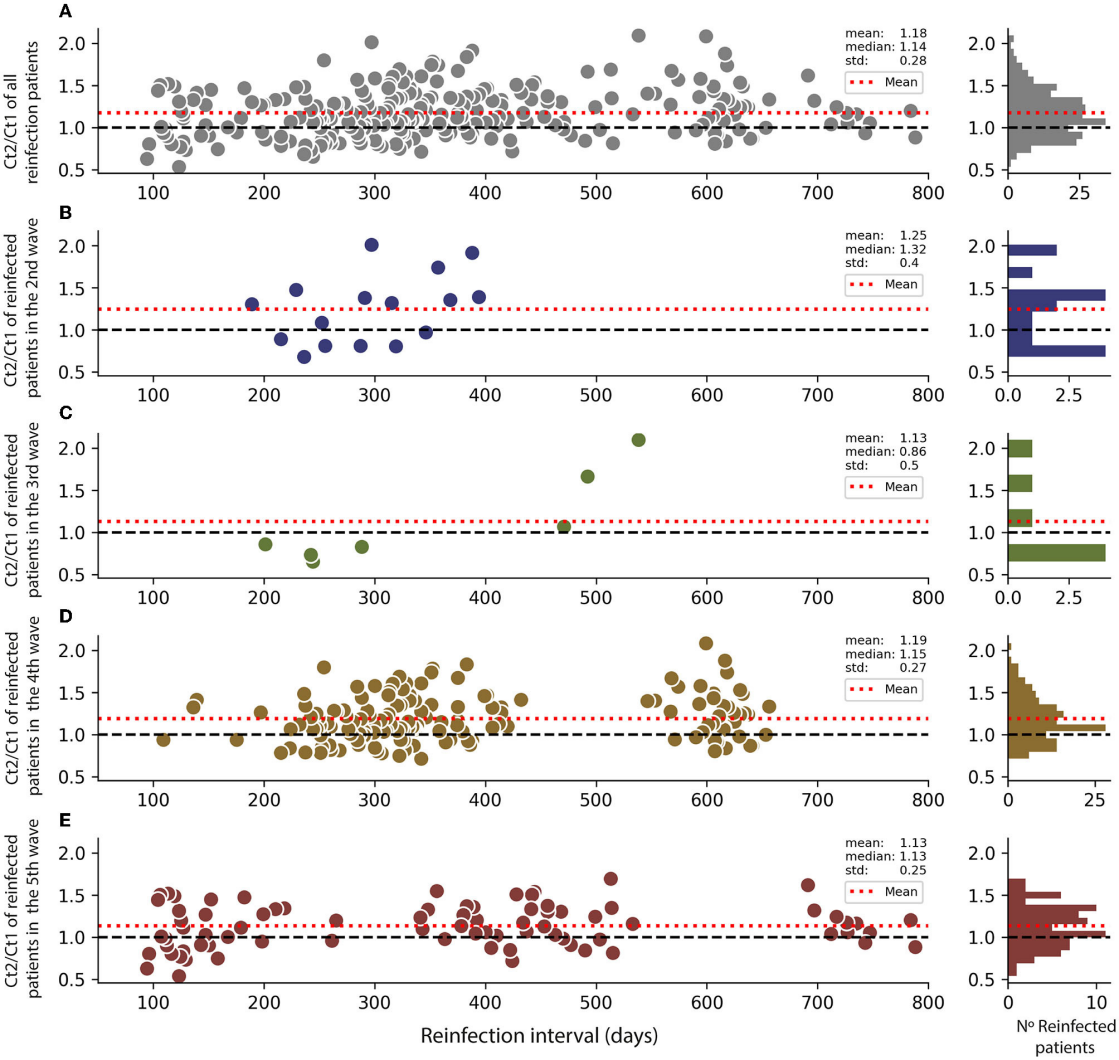
Variations of viral load in reinfected patients in different surges. Ct2 indicates the reinfection event, and Ct1 is the first infection. Ct values are the cycle threshold in RT-qPCR assay. **(A)** Ratio Ct2/Ct1 for all reinfection in the function of the reinfection interval. The red line shows the ratio average. A histogram of interval distribution is shown to the right. **(B)** Ratio Ct2/Ct1 for all reinfection during the second surge. **(C)** Ratio Ct2/Ct1 for all reinfection during the third surge. **(D)** Ratio Ct2/Ct1 for all reinfection during the fourth surge. **(E)** Ratio Ct2/Ct1 for all reinfection during the fifth surge.

The cycle threshold (Ct) value during the diagnosis of COVID-19 is closely related to the viral load of the infected patient (35), the risk of mortality during infection (36), and a greater capacity for the transmission and generation of contagion outbreaks (37). We evaluated the relationship between the Ct values of the second diagnosis (Ct2) against the initial infection (Ct1) to determine whether this reinfection was associated with a lower or higher viral load. Ratios of Ct2/Ct1 values from all intervals show a multimodal distribution with a mean of 1.18 and a median of 1.14 (Figure 5A). Ratios from patients reinfected in the second surge showed a higher mean and median (1.25 and 1.32, respectively, Figure 5B). In contrast, patients reinfected in the third and fourth surges have means closer to the value of overall reinfection patients Ct2/Ct1 ratio (Figures 5C, D). Patients reinfected in the fifth surge show a mean and median closer to 1, indicating similar Ct values in the second infection (Figure 5E). Since the histograms of the distribution of ratios show multimodal components, we analyzed each pair of surges. Average ratios higher than one were found in all pairs of waves, except surges 2–3, which are lower to one, and surges 4–5 are closer to one (Supplementary Figures 6, 7, Supplementary Table 4). In addition, all the pairs of waves showed a high percentage of values >1, including surges 4–5 (Supplementary Figure 7B). These results show that the behavior of ratios in surges 4–5 is unique and not shared with the other reinfections events that occurred in the fifth surge. Overall, these data indicate that the Ct2 values were higher in the second contagion in a population of patients, so the viral load in a reinfection event was mainly lower.

### 3.4. Overall reinfection analysis

In addition, we use an alternative analysis of reinfections independent of the date of occurrence without restricting surge dates. With this criterion, we found 283 patients with intervals between infections longer than 90 days, which occurred within surges and inter-surge periods. The cases that do not fall within waves are plotted in Figure 4B below the intervals between waves. The mean duration of all 283 intervals was 372.5; the median was 336, and the standard deviation was 171.4. The results of this cohort show a reinfection rate of 1.52 (95% CI: 1.34–1.70) per 100,000 inhabitants. These values are within the range obtained for the analysis using only reinfections between surges.

## 4. Discussion

Previous studies have documented the rate of reinfection processes in different countries and localities concerning the appearance of new variants of SARS-CoV-2 and various vaccination schemes. Gazit et al. (38) reported that in Israel, people who were naturally infected and then had a dose of vaccine significantly decreased the risk of reinfection by the Delta variant compared to people infected without any dose. A similar effect was determined by Malhotra et al. (39), in New Delhi, India, who indicated that unvaccinated patients had a 12.7% chance of reinfection compared to 1.6% of patients with two doses of vaccine in a cohort of 4,978 health workers. While Medić et al. (40), in a study carried out in Vojvodina, Serbia, highlighted that reinfections occurred mainly in women between the age of 30 and 39 years and 95% of the cases in patients without vaccination, while an event of reinfection occurred in 0.16% of people with complete vaccination (two doses) plus a booster dose. Reinfections increased significantly with the Omicron variant but with less severity than the Delta variant (40) when a similar situation was observed in South Africa (24). However, there are no reports on the analysis of the rate of reinfection concerning the variants, vaccination, and epidemiology in Chile. This study corresponds to a retrospective and descriptive analysis of reinfection events in a western zone of Santiago de Chile during the five surges of infections that have affected the country. A total of 578,670 tests were analyzed, corresponding to a total of 345,908 patients, of which 43,658 were diagnosed positive for COVID-19, at the laboratory of the University of Santiago de Chile (USACH), between 1 April 2020 and 31 July 2022. Reinfection events were considered >90 days after the first positive diagnosis, although viral persistence events have been reported even after 380 days (41), which are unusual and isolated cases. At the same time, even some reinfection criteria of ≥40 days have been reported (42). It was found that most of the reinfection events occurred during the Omicron propagation wave, where up to 0.772% of the total number of infections in the period was recorded. In comparison, the lowest prevalence of reinfection was recorded in the third surge, with the majority of the Delta variant. Although the Omicron variant is highly evasive of the immune system (43), our data on the reinfection rate were lower than that reported by the other studies. In this sense, e.g., the study by Nguyen et al. (44), in the city of Marseille, reported up to 6.8% reinfection, with an inclusion criterion of 90 days from the first positive diagnosis, where the Omicron variant was responsible. This difference can probably be explained due to the high vaccination rate in Chile (close to 80% with a full two-dose vaccination schedule and 70% with the first booster dose) when facing a surge of contagion from Omicron. Chile is one of the countries with the highest vaccination rate per 100 inhabitants in Latin America (45) and one of the countries that implemented vaccination the fastest worldwide (46). The policies implemented by the Government of Chile with the Ministry of Health generated a low incidence of reinfection. Even though, our data show a higher rate of reinfection in younger women than men, similar to finding reported by other studies (47, 48), even with an inclusion criterion of reinfection of >90 days after the first positive diagnosis. This could be reflected in less disease severity in contagion outbreaks since women are less likely to develop the severe disease than men (47). This higher rate of reinfections in women can be explained by women's more significant number of interpersonal contacts (49).

Regarding the number of intervals of days for reinfection to occur, an average of 358 days was found. This is similar to previously reported studies; for example, by Özüdogru et al. (50), who indicated an average of 361 days for reinfection, and Wilson (51), with an average of 343 days. The data obtained by the University of Santiago (USACH) were closely related to what was reported by the rest of the M.R., even up to the fifth wave of infections in July 2022. Therefore, since M.R. involves 40% of the total population of Chile, we could suggest that our data could closely represent the behavior of the rate of reinfection in the whole of Chile. In this sense, since patients' clinical history is not public, we speculate that reinfections would tend to represent a less severe disease (52–54) and lower transmissibility (55). In addition, the low viral load observed in patients reinfected by each contagion may be related to the efficacy of mass vaccination in Chile or simply to the immunity conferred after a natural infection by Omicron, as previously seen (24).

Our study has some limitations that are important to highlight. First, it did not differentiate the involvement of reinfection after natural, hybrid, or vaccination-only immunization between the RT-qPCR tests of the patients analyzed. These different ways of generating immunity in a patient could affect a reinfection event since not all yield the same protection capacity (56), resulting in more or fewer days between one infection or another. Our data do not consider cases identified by rapid antigen tests. These tests were used massively during the waves of Omicron. This reduced the proportion of reinfection cases determined by RT-qPCR in our dataset. Rapid antigen tests in Chile represent 18% of the total tests carried out during the pandemic (57); therefore, our data may be underrepresented. However, our results show that the highest reinfection rate occurred with Omicron's arrival, which is consistent with other reports (40, 50).

On the other hand, the relationship between the vaccination rate and the number of reinfection events occurring in a period was only descriptive, because there is no information on the vaccination status of the study patients. At the same time, there was no discrimination between the different types of vaccines administered (58), which generated different degrees of protection efficacy against a new SARS-CoV-2 infection. Finally, no difference was made between the Omicron subvariants, which can cause reinfections with different time intervals (59). However, despite these limitations, our study indicates a rate of reinfection in Chile, similar to research from other countries, supported by extensive RT-qPCR test data.

This is the first retrospective report on the prevalence of reinfection in Chile, with the largest dataset of patients analyzed to date, giving our analyses greater robustness. These data could be helpful and of particular interest to government authorities for continuously implementing public health policies to control the pandemic and for epidemiological groups with a greater predisposition to reinfection. Although reinfection seems to be a rare process, there is a probability that it can occur, even in populations with a high vaccination rate. Our study demonstrates the need for epidemiological monitoring of SARS-CoV-2 since an increase in reinfection rates in a locality could account for the appearance of new, more transmissible, and evasive variants.

## Data availability statement

The raw data supporting the conclusions of this article will be made available by the authors, without undue reservation.

## Ethics statement

The studies involving human participants were reviewed and approved by Ethics Committee of the University of Santiago of Chile (No. 226/2021) and the Scientific Ethical Committee of the Central Metropolitan Health Service, Ministry of Health, Government of Chile (No. 370/2021), following Chilean legislation. Written informed consent for participation was not required for this study in accordance with the national legislation and the institutional requirements.

## Author contributions

CA-C and FER-L: conceptualization. PR and CA-C: methodology and data curation. AMS, FER-L, and MI: validation. PR: formal analysis. VB, AI-M, MV, RL, EV-V, and AM-T: investigation. CA-C, CB-A, MI, FER-L, and AMS: resources. CA-C and CB-A: writing—original draft preparation. CA-C, LM, FER-L, PR, CB-A, and VB: writing—review and editing. DV and MI: visualization. CA-C, FER-L, and AMS: supervision. FER-L and AMS: project administration and funding acquisition. All authors have read and agreed to the published version of the manuscript.

## References

[1] World Health Organization. WHO Coronavirus (COVID-19) Dashboard. World Health Organization (2023). Available online at: https://covid19.who.int/ (accessed June 22, 2023).

[2] Kojima N Klausner JD. Protective immunity after recovery from SARS-CoV-2 infection. Lancet Infect Dis. (2022) 22:12–4. 10.1016/S1473-3099(21)00676-9 34762853 PMC8575467

[3] Yu Y Esposito D Kang Z Lu J Remaley AT De Giorgi V. mRNA vaccine-induced antibodies more effective than natural immunity in neutralizing SARS-CoV-2 and its high affinity variants. Sci Rep. (2022) 12:2628. 10.1038/s41598-022-06629-2 35173254 PMC8850441

[4] Iketani S Liu L Guo Y Liu L Chan JF-W Huang Y. Antibody evasion properties of SARS-CoV-2 Omicron sublineages. Nature. (2022) 604:553–6. 10.1038/s41586-022-04594-4 35240676 PMC9021018

[5] Wilkins JT Hirschhorn LR Gray EL Wallia A Carnethon M Zembower TR. Serologic status and SARS-CoV-2 infection over 6 months of follow up in healthcare workers in Chicago: a cohort study. Infect Control Hosp Epidemiol. (2022) 43:1207–15. 10.1017/ice.2021.367 34369331 PMC8438416

[6] De Giorgi V West KA Henning AN Chen LN Holbrook MR Gross R. Naturally acquired SARS-CoV-2 immunity persists for up to 11 months following infection. J Infect Dis. (2021) 224:1294–304. 10.1093/infdis/jiab295 34089610 PMC8195007

[7] Feikin DR Higdon MM Abu-Raddad LJ Andrews N Araos R Goldberg Y. Duration of effectiveness of vaccines against SARS-CoV-2 infection and COVID-19 disease: results of a systematic review and meta-regression. Lancet. (2022) 399:924–44. 10.1016/S0140-6736(22)00152-0 35202601 PMC8863502

[8] Nabel KG Clark SA Shankar S Pan J Clark LE Yang P. Structural basis for continued antibody evasion by the SARS-CoV-2 receptor binding domain. Science. (2022) 375:eabl6251. 10.1126/science.abl6251 34855508 PMC9127715

[9] World Health Organization. Tracking SARS-CoV-2 Variants. World Health Organization (2023). Available online at: https://www.who.int/en/activities/tracking-SARS-CoV-2-variants (accessed May 3, 2023).

[10] Jassat W Abdool Karim SS Mudara C Welch R Ozougwu L Groome MJ. Clinical severity of COVID-19 in patients admitted to hospital during the omicron wave in South Africa: a retrospective observational study. Lancet Glob Health. (2022) 10:e961–9. 10.1016/S2214-109X(22)00114-0 35597249 PMC9116895

[11] Wise J. Covid-19: Omicron sub variants driving new wave of infections in UK. BMJ. (2022) 377:o1506. 10.1136/bmj.o1506 35724993

[12] Sigal A Milo R Jassat W. Estimating disease severity of Omicron and Delta SARS-CoV-2 infections. Nat Rev Immunol. (2022) 22:267–9. 10.1038/s41577-022-00720-5 35414124 PMC9002222

[13] Adjei S Hong K Molinari N-AM Bull-Otterson L Ajani UA Gundlapalli AV. Mortality risk among patients hospitalized primarily for COVID-19 during the omicron and delta variant pandemic periods — United States, April 2020–June 2022. MMWR Morb Mortal Wkly Rep. (2022) 71:1182–9. 10.15585/mmwr.mm7137a4 36107788 PMC9484808

[14] Ke H Chang MR Marasco WA. Immune evasion of SARS-CoV-2 Omicron subvariants. Vaccines. (2022) 10:1545. 10.3390/vaccines10091545 36146623 PMC9501521

[15] Jiang X-L Zhu K-L Wang X-J Wang G-L Li Y-K He X-J. Omicron BQ1 and BQ11 escape neutralisation by omicron subvariant breakthrough infection. Lancet Infect Dis. (2023) 23:28–30. 10.1016/S1473-3099(22)00805-2 36543471 PMC9762743

[16] Pilz S Theiler-Schwetz V Trummer C Krause R Ioannidis JPA. SARS-CoV-2 reinfections: overview of efficacy and duration of natural and hybrid immunity. Environ Res. (2022) 209:112911. 10.1016/j.envres.2022.112911 35149106 PMC8824301

[17] Acuña-Castillo C Vidal M Inostroza-Molina A Vallejos-Vidal E Luraschi R Figueroa M. First identification of reinfection by a genetically different variant of SARS-CoV-2 in a homeless person from the metropolitan area of Santiago, Chile. J Environ Public Health. (2022) 2022:1–6. 10.1155/2022/3859071 35528635 PMC9068328

[18] Tillett RL Sevinsky JR Hartley PD Kerwin H Crawford N Gorzalski A. Genomic evidence for reinfection with SARS-CoV-2: a case study. Lancet Infect Dis. (2021) 21:52–8. 10.1016/S1473-3099(20)30764-7 33058797 PMC7550103

[19] Pérez-Lago L Kestler M Sola-Campoy PJ Rodriguez-Grande C Flores-García RF Buenestado-Serrano S. SARS-CoV-2 superinfection and reinfection with three different strains. Transbound Emerg Dis. (2022) 69:3084–9. 10.1111/tbed.14352 34687493 PMC8662055

[20] Rosenberg M Chen C Golzarri-Arroyo L Carroll A Menachemi N Ludema C. SARS-CoV-2 reinfections in a US university setting, Fall 2020 to Spring 2021. BMC Infect Dis. (2022) 22:592. 10.1186/s12879-022-07578-x 35787250 PMC9252534

[21] Ringlander J Olausson J Nyström K Härnqvist T Jakobsson HE Lindh M. Recurrent and persistent infection with SARS-CoV-2 – epidemiological data and case reports from Western Sweden, 2020. Infect Dis. (2021) 53:900–7. 10.1080/23744235.2021.1957143 34308755

[22] Yang SL Teh HS Suah JL Husin M Hwong WY. SARS-CoV-2 in Malaysia: a surge of reinfection during the predominantly Omicron period. Lancet Reg Health West Pac. (2022) 26:100572. 10.1016/j.lanwpc.2022.100572 36060536 PMC9417595

[23] Eythorsson E Runolfsdottir HL Ingvarsson RF Sigurdsson MI Palsson R. Rate of SARS-CoV-2 reinfection during an omicron wave in Iceland. JAMA Netw Open. (2022) 5:e2225320. 10.1001/jamanetworkopen.2022.25320 35921113 PMC9350711

[24] Pulliam JRC van Schalkwyk C Govender N von Gottberg A Cohen C Groome MJ. Increased risk of SARS-CoV-2 reinfection associated with emergence of Omicron in South Africa. Science. (2022) 376:eabn4947. 10.1126/science.abn4947 35289632 PMC8995029

[25] Barrera-Avalos C Luraschi R Vallejos-Vidal E Figueroa M Arenillas E Barría D. Analysis by real-time PCR of five transport and conservation mediums of nasopharyngeal swab samples to COVID-19 diagnosis in Santiago of Chile. J Med Virol. (2022) 94:1167–74. 10.1002/jmv.27446 34755352 PMC8662110

[26] Luraschi R Barrera-Avalos C Vallejos-Vidal E Alarcón J Mella-Torres A Hernández F. The comparative analysis of two RT-qPCR kits for detecting SARS-CoV-2 reveals a higher risk of false-negative diagnosis in samples with high quantification cycles for viral and internal genes. Can J Infect Dis Med Microbiol. (2022) 2022:1–10. 10.1155/2022/2594564 35812012 PMC9259548

[27] Montes-González JA Zaragoza-Jiménez CA Antonio-Villa NE Fermín-Martínez CA Ramírez-García D Vargas-Vázquez A. Protection of hybrid immunity against SARS-CoV-2 reinfection and severe COVID-19 during periods of Omicron variant predominance in Mexico. Front Public Health. (2023) 11:1146059. 10.3389/fpubh.2023.1146059 37081954 PMC10110947

[28] The Pan American Health Organization/World Health Organization. (PAHO/WHO). Interim Guidelines for Detecting Cases of Reinfection by SARSCoV-2. Washington, DC: Pan American Health Organization/World Health Organization (2020). Available online at: https://www.paho.org/en/documents/interim-guidelines-detecting-cases-reinfection-sars-cov-2 (accessed April15, 2023).

[29] Centers for Disease Control Prevention. (CDC). What is COVID-19 Reinfection? (2023). Available online at: https://www.cdc.gov/coronavirus/2019-ncov/your-health/reinfection.html (accessed April 15, 2023).

[30] Ministerio de Ciencia Tecnología Conocimiento e Innovación G of C. Base de Datos COVID-19. (2022). Available online at: https://www.minciencia.gob.cl/covid19/ (accessed October 10, 2022).

[31] Instituto de Salud Pública de Chile Government of Chile. Vigilancia Genómica SARS-Cov2 ISP. (2022). Available online at: https://vigilancia.ispch.gob.cl/app/varcovid (accessed October 5, 2022).

[32] Walker AS Pritchard E House T Robotham JV Birrell PJ Bell I. Ct threshold values, a proxy for viral load in community SARS-CoV-2 cases, demonstrate wide variation across populations and over time. Elife. (2021) 10:e64683. 10.7554/eLife.64683 34250907 PMC8282332

[33] Hansen CH Michlmayr D Gubbels SM Mølbak K Ethelberg S. Assessment of protection against reinfection with SARS-CoV-2 among 4 million PCR-tested individuals in Denmark in 2020: a population-level observational study. Lancet. (2021) 397:1204–12. 10.1016/S0140-6736(21)00575-4 33743221 PMC7969130

[34] Nguyen NN Houhamdi L Hoang VT Delerce J Delorme L Colson P. SARS-CoV-2 reinfection and COVID-19 severity. Emerg Microbes Infect. (2022) 11:894–901. 10.1080/22221751.2022.2052358 35264078 PMC8942490

[35] Luraschi R Santibáñez Á Barrera-Avalos C Vallejos-Vidal E Mateluna-Flores C Alarcón J. Evaluation and comparison of the sensitivity of three commercial RT-qPCR kits used for the detection of SARS-CoV-2 in Santiago, Chile. Front Public Health. (2022) 10:1010336. 10.3389/fpubh.2022.1010336 36518569 PMC9742446

[36] Rico-Caballero V Fernández M Hurtado JC Marcos MA Cardozo C Albiach L. Impact of SARS-CoV-2 viral load and duration of symptoms before hospital admission on the mortality of hospitalized COVID-19 patients. Infection. (2022) 50:1321–8. 10.1007/s15010-022-01833-8 35562568 PMC9105593

[37] Puhach O Meyer B Eckerle I. SARS-CoV-2 viral load and shedding kinetics. Nat Rev Microbiol. (2022) 21:147–61. 10.1038/s41579-022-00822-w 36460930 PMC9716513

[38] Gazit S Shlezinger R Perez G Lotan R Peretz A Ben-Tov A. The incidence of SARS-CoV-2 reinfection in persons with naturally acquired immunity with and without subsequent receipt of a single dose of BNT162b2 vaccine. Ann Intern Med. (2022) 175:674–81. 10.7326/M21-4130 35157493 PMC8855786

[39] Malhotra S Mani K Lodha R Bakhshi S Mathur VP Gupta P. SARS-CoV-2 Reinfection rate and estimated effectiveness of the inactivated whole virion vaccine BBV152 against reinfection among health care workers in New Delhi, India. JAMA Netw Open. (2022) 5:e2142210. 10.1001/jamanetworkopen.2021.42210 34994793 PMC8742193

[40] Medić S Anastassopoulou C Lozanov-Crvenković Z Vuković V Dragnić N Petrović V. Risk and severity of SARS-CoV-2 reinfections during 2020–2022 in Vojvodina, Serbia: a population-level observational study. Lancet Reg Health Eur. (2022) 20:100453. 10.1016/j.lanepe.2022.100453 35791336 PMC9246704

[41] Acuña-Castillo C Maisey K Vidal M Barrera-Avalos C Inostroza-Molina A Luraschi R. Genomic evidence suggests viral persistence of SARS-CoV-2 for 386 days in health worker: a case report from Santiago of Chile. Infect Dis Rep. (2022) 14:971–8. 10.3390/idr14060096 36547242 PMC9778366

[42] Santiago-Espinosa O Prieto-Torres ME Cabrera-Gaytán DA. Laboratory-confirmed SARS-CoV-2 reinfection in the population treated at social security. Respir Med Case Rep. (2021) 34:101493. 10.1016/j.rmcr.2021.101493 34395189 PMC8351271

[43] Willett BJ Grove J MacLean OA Wilkie C De Lorenzo G Furnon W. SARS-CoV-2 Omicron is an immune escape variant with an altered cell entry pathway. Nat Microbiol. (2022) 7:1161–79. 10.1038/s41564-022-01143-7 35798890 PMC9352574

[44] Nguyen NN Houhamdi L Hoang VT Stoupan D Fournier P-E Raoult D. High rate of reinfection with the SARS-CoV-2 Omicron variant. J Infect. (2022) 85:174–211. 10.1016/j.jinf.2022.04.034 35472367 PMC9033627

[45] Statista. Number of COVID-19 Vaccination Doses per-100 Population Administered in Latin America and the Caribbean. (2023). Available online at: http://www.statista.com/statistics/1194813/latin-america-covid-19-vaccination-ratecountry (accessed January 15, 2023).

[46] Castillo C Villalobos Dintrans P Maddaleno M. The successful COVID-19 vaccine rollout in Chile: factors and challenges. Vaccine X. (2021) 9:100114. 10.1016/j.jvacx.2021.100114 34518818 PMC8425670

[47] Lawandi A Warner S Sun J Demirkale CY Danner RL Klompas M. Suspected Severe Acute Respiratory Syndrome Coronavirus 2 (SARS-COV-2) reinfections: incidence, predictors, and healthcare use among patients at 238 US Healthcare Facilities, 1 June 2020 to 28 February 2021. Clin Infect Dis. (2022) 74:1489–92. 10.1093/cid/ciab671 34351392 PMC8436398

[48] Flacco ME Soldato G Acuti Martellucci C Di Martino G Carota R Caponetti A. Risk of SARS-CoV-2 reinfection 18 months after primary infection: population-level observational study. Front Public Health. (2022) 10:884121. 10.3389/fpubh.2022.884121 35586006 PMC9108359

[49] Doerre A Doblhammer G. The influence of gender on COVID-19 infections and mortality in Germany: insights from age- and gender-specific modeling of contact rates, infections, and deaths in the early phase of the pandemic. PLoS ONE. (2022) 17:e0268119. 10.1371/journal.pone.0268119 35522614 PMC9075634

[50] Özüdogru O Bahçe YG Acer Ö. SARS CoV-2 reinfection rate is higher in the Omicron variant than in the Alpha and Delta variants. Ir J Med Sci. (2022) 192:751–6. 10.1007/s11845-022-03060-4 35711013 PMC9203229

[51] Wilson C. How quickly can you catch covid-19 again? New Sci. (2022) 254:9. 10.1016/S0262-4079(22)00824-7 35603060 PMC9106377

[52] de Magalhães JJF Mendes RPG da Silva CTA da Silva SJR Guarines KM Pena L. Epidemiological and clinical characteristics of the first 557 successive patients with COVID-19 in Pernambuco state, Northeast Brazil. Travel Med Infect Dis. (2020) 38:101884. 10.1016/j.tmaid.2020.101884 32971239 PMC7522369

[53] Tan L Kang X Ji X Li G Wang Q Li Y. Validation of predictors of disease severity and outcomes in COVID-19 patients: a descriptive and retrospective study. Med. (2020) 1:128–38.e3. 10.1016/j.medj.2020.05.002 32838352 PMC7235581

[54] Liu Y Yan L-M Wan L Xiang T-X Le A Liu J-M. Viral dynamics in mild and severe cases of COVID-19. Lancet Infect Dis. (2020) 20:656–7. 10.1016/S1473-3099(20)30232-2 32199493 PMC7158902

[55] Dadras O Afsahi AM Pashaei Z Mojdeganlou H Karimi A Habibi P. The relationship between COVID-19 viral load and disease severity: a systematic review. Immun Inflamm Dis. (2022) 10:e580. 10.1002/iid3.580 34904379 PMC8926507

[56] Huang L Lai FTT Yan VKC Cheng FWT Cheung CL Chui CSL. Comparing hybrid and regular COVID-19 vaccine-induced immunity against the Omicron epidemic. NPJ Vaccines. (2022) 7:162. 10.1038/s41541-022-00594-7 36522341 PMC9753877

[57] Ministry of Health Government of Chile. COVID-19 Official Numbers. (2023). Available online at: https://www.gob.cl/pasoapaso/cifrasoficiales/ (accessed May 2, 2023).

[58] Graña C Ghosn L Evrenoglou T Jarde A Minozzi S Bergman H. Efficacy and safety of COVID-19 vaccines. Cochrane Database Syst Rev. (2022) 2023:CD015477. 10.1002/14651858.CD015477 36473651 PMC9726273

[59] Nguyen NN Houhamdi L Delorme L Colson P Gautret P. Reinfections with different SARS-CoV-2 Omicron subvariants, France. Emerg Infect Dis. (2022) 28:2341–3. 10.3201/eid2811.221109 36150518 PMC9622229

