# Supplementary material for: An ecological study on reinfection rates using a large dataset of RT-qPCR tests for SARS-CoV-2 in Santiago of Chile
Source: Front Public Health. 2023 Jul 10;11:1191377. doi: 10.3389/fpubh.2023.1191377 (PMC10364051; doi:10.3389/fpubh.2023.1191377)
Supplement: Supplementary file 1 [file Data_Sheet_1.pdf]

frontiers

Supplementary Material

An ecological study on reinfection rates using a large data set of RT-qPCR tests for SARS-CoV-2 in Chile

Claudio Acuña-Castillo[1,2] §, Carlos Barrera-Avalos[2]§, Vivienne C. Bachelet[3], Luis A. Milla[4], Ailén Inostroza-Molina[1], Mabel Vidal[5], Roberto Luraschi[2], Eva Vallejos-Vidal[2], Andrea Mella-Torres[2], Daniel Valdés[1], Felipe E. Reyes-López[2], Mónica Imarai[1,2]*, Patricio Rojas[1]* and Ana M. Sandino[1,2]*.

[1] Departamento de Biología, Facultad de Química y Biología, Universidad de Santiago de Chile, Santiago, Chile
[2] Centro de Biotecnología Acuícola, Facultad de Química y Biología, Universidad de Santiago de Chile, Santiago, Chile
[3] Escuela de Medicina, Facultad de Ciencias Médicas, Universidad de Santiago de Chile, Santiago, Chile
[4] Centro de Investigaciones Biomédicas y Aplicadas, Escuela de Medicina, Facultad de Ciencias Médicas, Universidad de Santiago de Chile
[5] Department of Computer Science, University of Concepcion, Concepción, Chile

§These authors have contributed equally to this work.

Running title: Rate of reinfection by SARS-CoV-2 in Chile

*** Correspondence:**

Laboratorio de Virología, Centro de Biotecnología Acuícola, Departamento de Biología, Facultad de Química y Biología, Universidad de Santiago de Chile, Alameda Libertador Bernardo O'Higgins 3363, Estación Central, Correo 40, box 33, Santiago, Chile (Mónica Imarai, monica.imarai@usach.cl; Patricio Rojas, patricio.rojas.m@usach.cl and Ana M. Sandino, ana.sandino@usach.cl).

Supplementary Figures

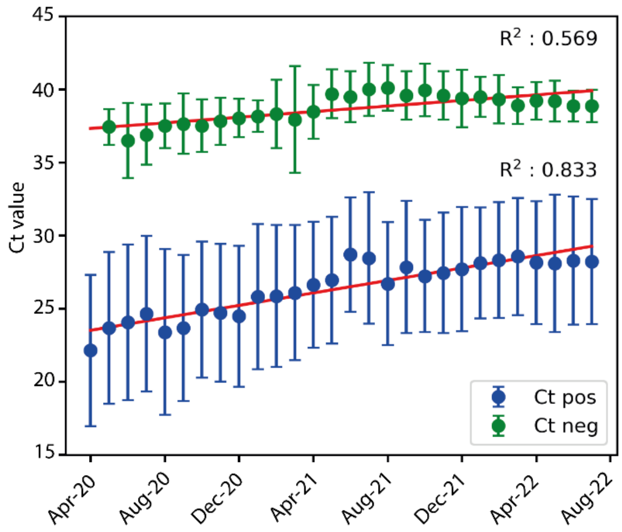

Supplementary Figure 1. Monthly variation of Ct values for positive and negative tests. Linear regression fit shows an increase in Ct during the pandemic compared to negative tests. Data is shown as mean (circles) and standard deviation (bars). Fit parameters for positive tests are: slope = 0.213, intercept = 23.29. Fit parameters for negative tests are: slope = 0.096, intercept = 37.22

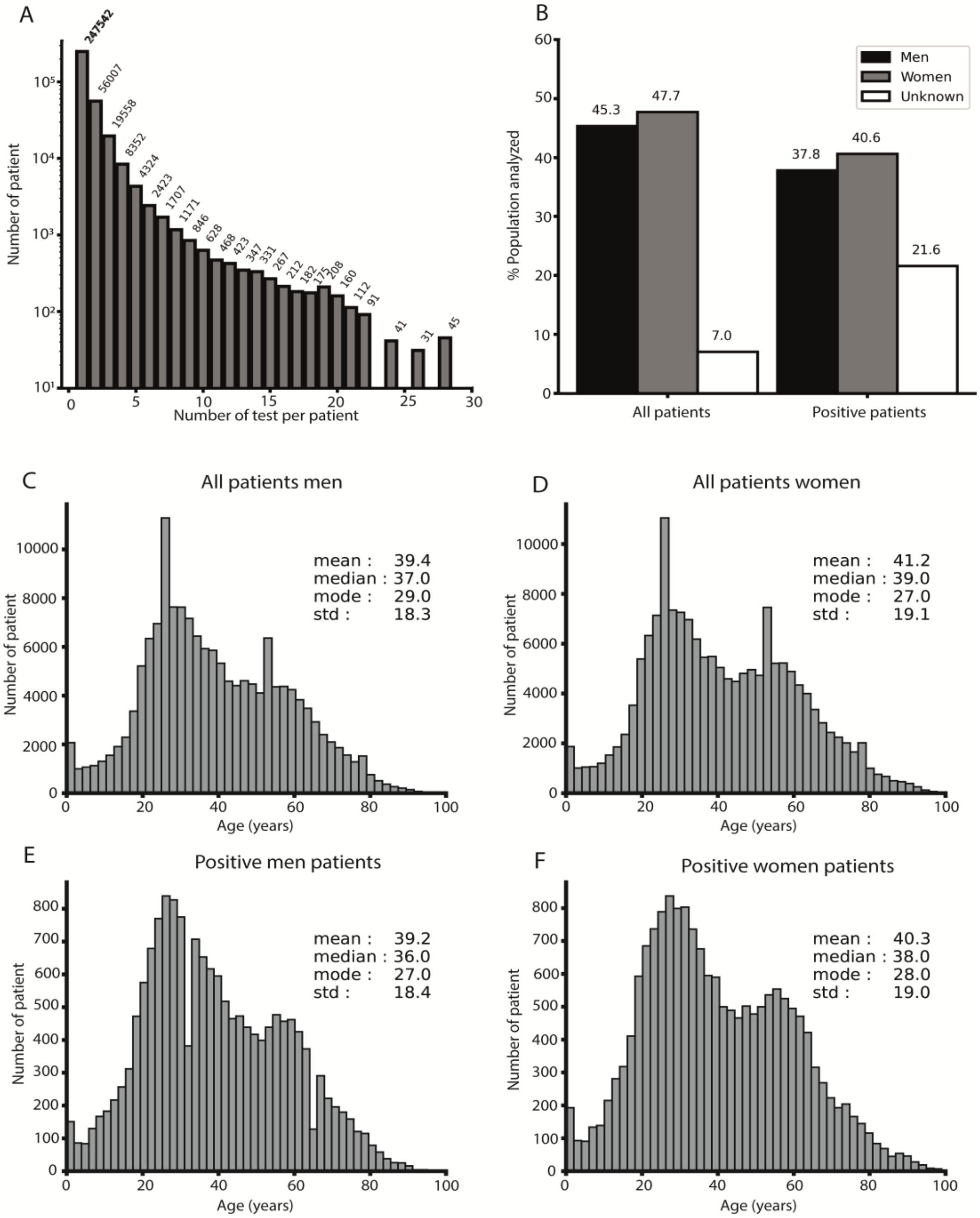

Supplementary Figure 2. Number of tests per patient and sex distribution. A, histogram of the number of tests per person to all patients in the dataset, in logarithmic scale. **B,** percentage of sex for all patients and positive patients. **C** and **D**, age, and distribution of all patients -men and women- participating in the study, respectively. **E** and **F**, age, and distribution of only positive patients -men and women- participating in the study, respectively.

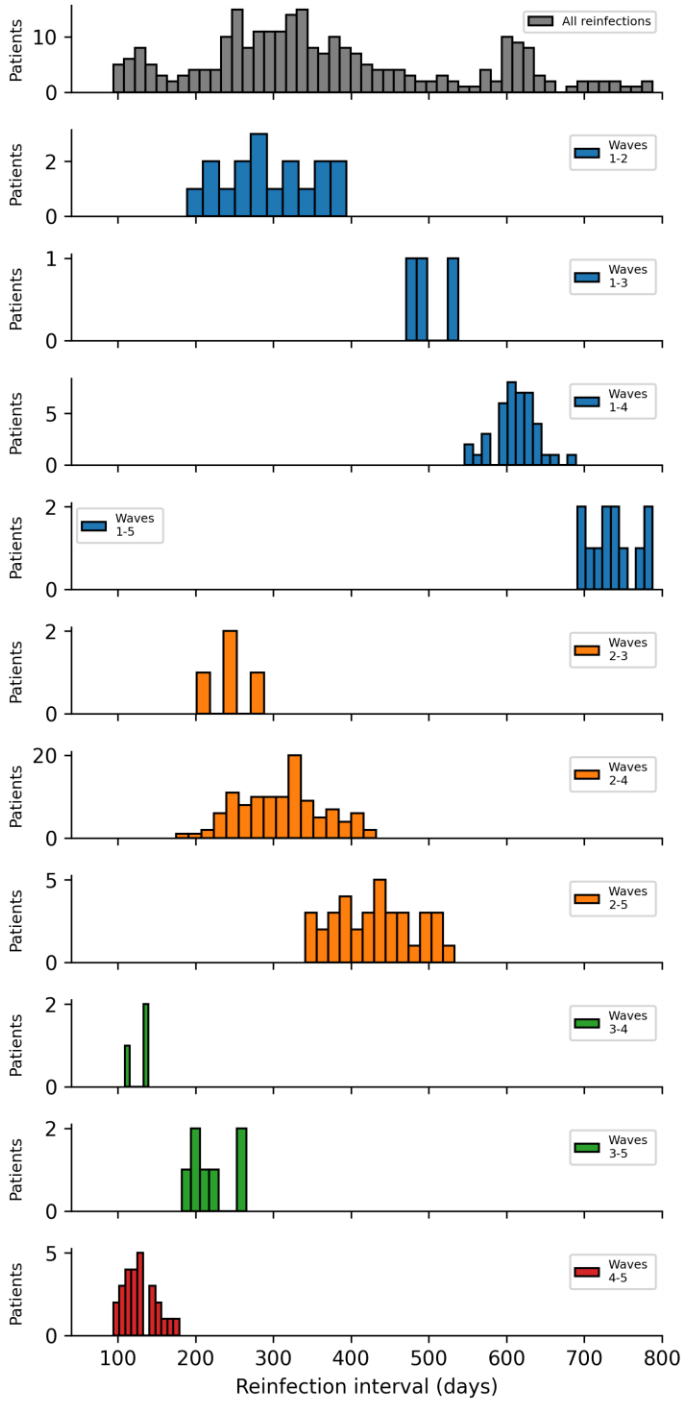

Supplementary Figure 3. Distribution of intervals between infections for all pairs of waves. The top line is the histogram for all reinfections. The following lines are the histograms for intervals for each of the waves. Colors show the surge with the first positive test (first wave: blue, second wave: orange, third wave: green, fourth wave: red).

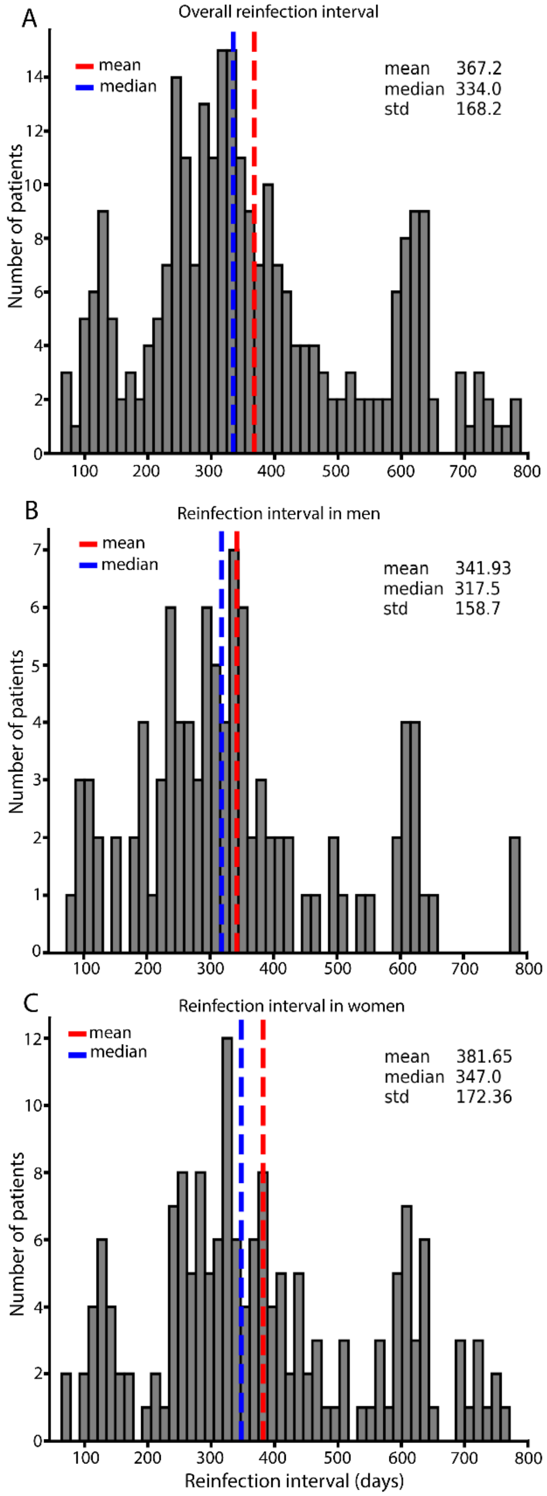

Supplementary Figure 4. Distribution of intervals between infections for sex. **A**, histogram of the distribution of all reinfected patients. **B**, histogram of the distribution of men. **C**, histogram of the distribution for women

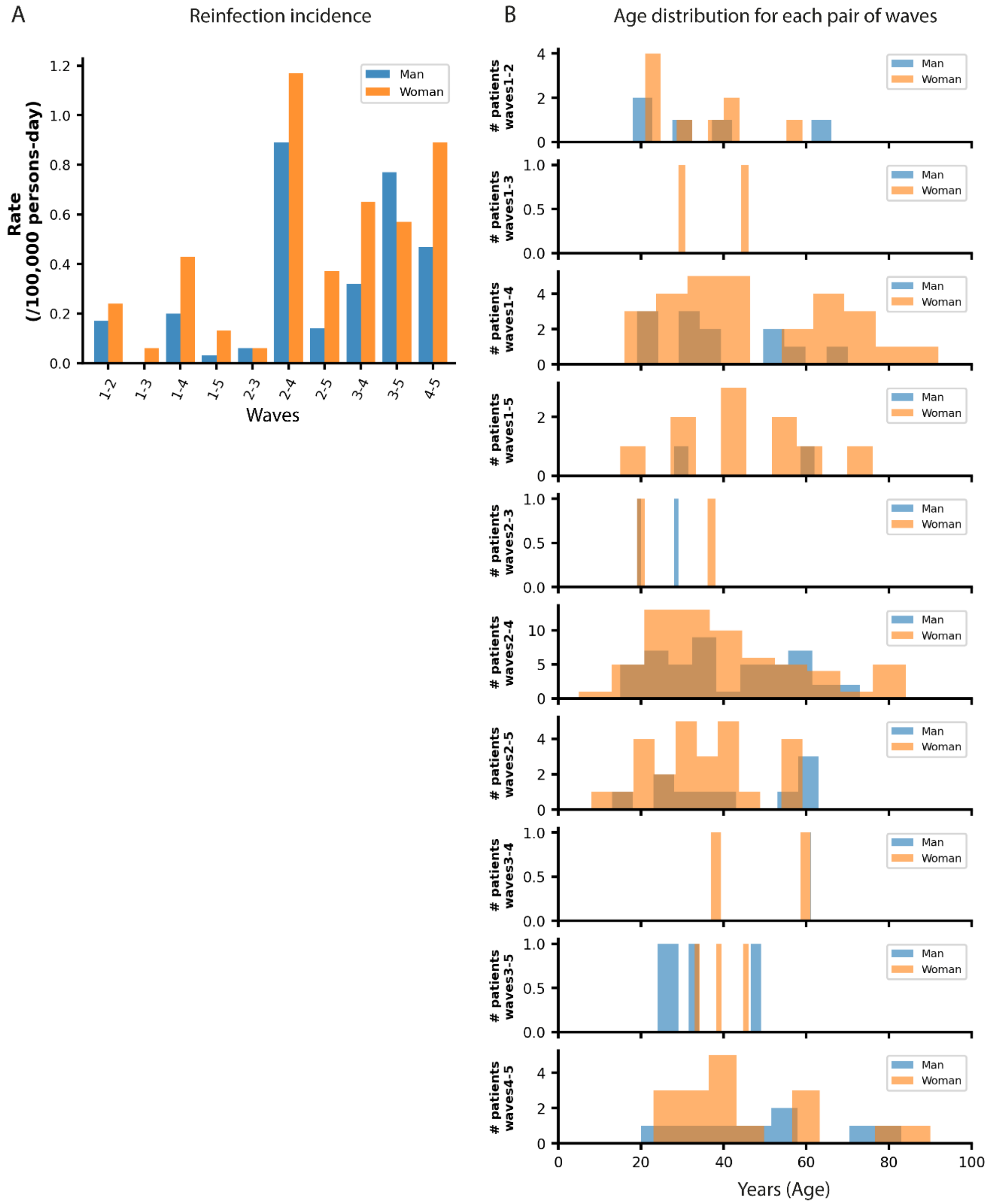

Supplementary Figure 5. Reinfections in women and men patients. A, Rate of reinfected men and woman in each of the pairs of surges studied. **B**, histogram of the age distribution for each pair of waves for man (light orange) and woman (light green).

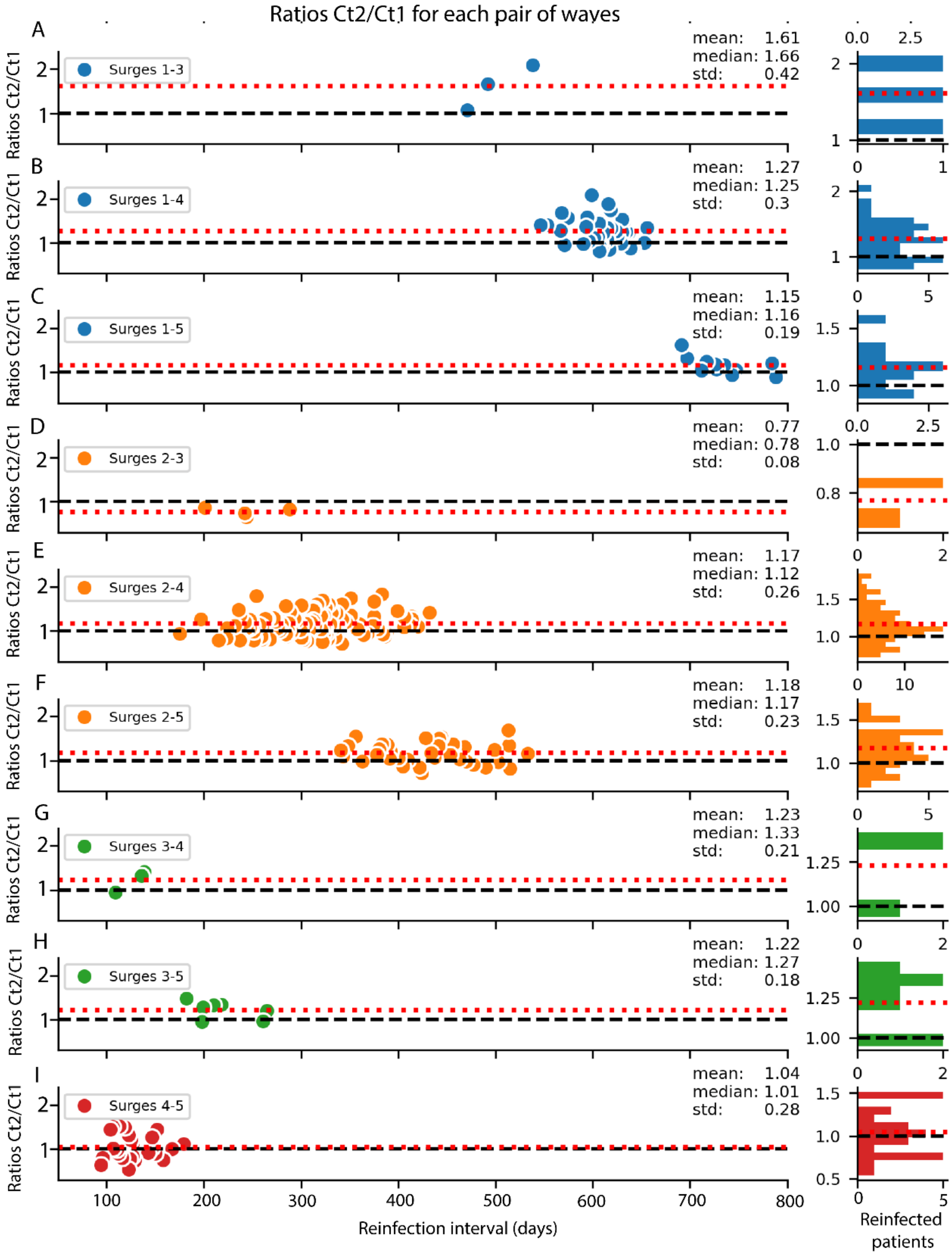

Supplementary Figure 6. Ratios Ct2/Ct1 for each pair of waves. Circles represent the ratio Ct2/Ct1 in function of the duration of reinfection interval for each patient. The red dotted line shows the average ratios for each pair of waves. A histogram of distribution or ratios was calculated for each pair of waves at a different scale in the y-axis to compare data distribution with the unity (black dashed line)

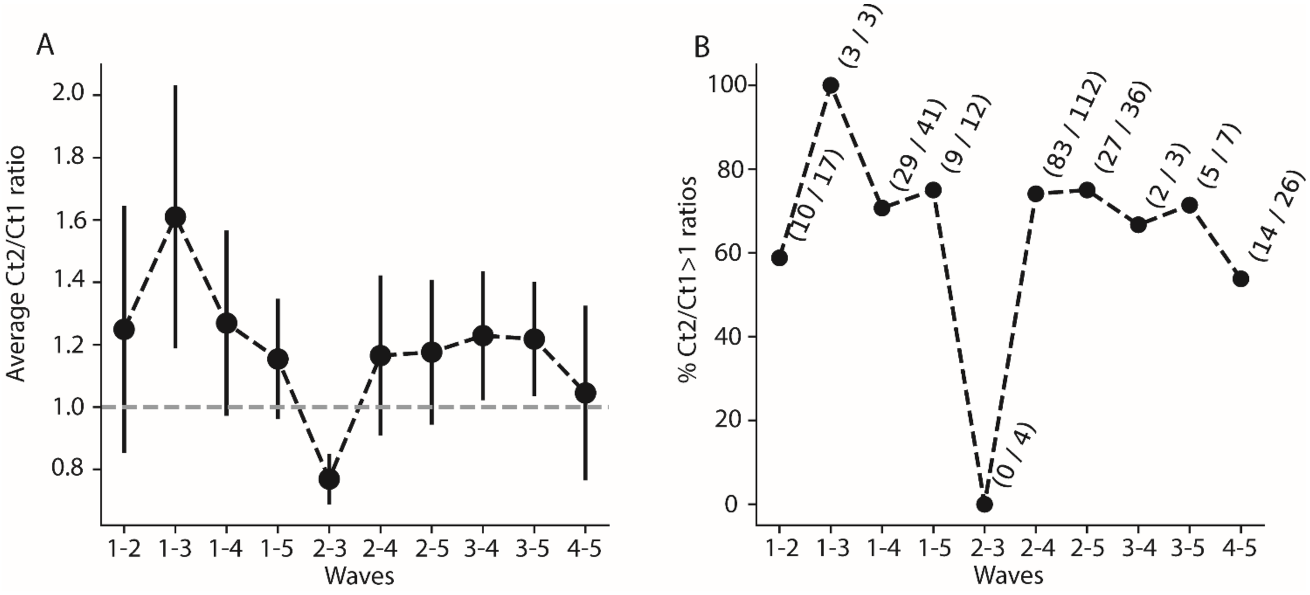

Supplementary Figure 7. Average ratios Ct2/Ct1 for each pair of surges. A, Average ratio, and standard deviation for each pair of surges. **B**, percentage of ratios higher than 1 for each pair of surges. Numbers in parenthesis indicate the number of ratios higher than one over the total number of ratios.

Supplementary Tables

Supplementary Table Nº 1. Statistical parameters of reinfection intervals ending in second, third, fourth and fifth surges.

Surge of reinfection	Mean	Median	Std	N
2nd	294.65	291	59.79	17 (6.4 %)
3rd	353.71	288	130.38	7 (2.6 %)
4th	386.25	331	144.78	156 (58.9 %)
5th	361.21	383	209.58	81 (30.6 %)

Supplementary Table Nº2. Descriptive statistics of reinfection intervals from all the surges.

Dataset	Mean	Median	Std	N
All intervals	371.6	335	165.5	261
Surges 1-2	294.6	291	59.8	17 (6.4 %)
Surges 1-3	500.3	492	28	3 (1.1 %)
Surges 1-4	611.3	615	27.7	41 (15.5 %)
Surges 1-5	736.5	731	30.4	12 (4.5 %)
Surges 2-3	243.8	243	30.8	4 (1.5 %)
Surges 2-4	310.8	312.5	53.8	112 (42.3 %)
Surges 2-5	432.2	435.5	52.8	36 (13.6 %)
Surges 3-4	128	136	13.5	3 (1.1 %)
Surges 3-5	219	210	29.7	7 (2.6 %)
Surges 4-5	127.9	124.5	21.3	26 (9.8 %)

Supplementary Table Nº3. Descriptive statistics of reinfection intervals starting in first, second, third and fourth surges.

Surge of infection	Mean	Median	Std	N
1st	553.6	607	156.7	73 (27,5%)
2nd	337.8	329	75.5	152 (57.4 %)
3rd	191.7	198.5	49.1	10 (3.8 %)
4th	127.9	124.5	21.3	26 (9.8 %)

Supplementary Table Nº4. Descriptive statistics of ratios Ct2/Ct1 for reinfections

Pair of surges	Mean	Median	Std	Proportion ratios >1	Number ratios >1	N
1-2	1.25	1.32	0.4	0.59	10	17
1-3	1.61	1.66	0.42	1	3	3
1-4	1.27	1.26	0.3	0.71	29	41
1-5	1.15	1.16	0.19	0.75	9	12
2-3	0.77	0.78	0.08	0	0	4
2-4	1.17	1.12	0.26	0.74	83	112
2-5	1.18	1.17	0.23	0.75	27	36
3-4	1.23	1.33	0.21	0.67	2	3
3-5	1.22	1.27	0.18	0.71	5	7
4-5	1.05	1.01	0.28	0.54	14	26
